# Body Size Shifts in Philippine Reef Fishes: Interfamilial Variation in Responses to Protection

**DOI:** 10.3390/biology3020264

**Published:** 2014-03-31

**Authors:** Robert Y. Fidler, Aileen Maypa, Dean Apistar, Alan White, Ralph G. Turingan

**Affiliations:** 1 Florida Institute of Technology, Melbourne, FL 32901, USA; E-Mail: turingan@fit.edu; 2 Coastal Conservation and Education Foundation, Banilad, Cebu City 6000, Philippines; E-Mails: aimaypa@yahoo.com (A.M.); deaniro@yahoo.com (D.A.); 3 University of Hawai’i at Mãnoa, Honolulu, HI 96822, USA; 4 The Nature Conservancy Global Marine Initiative, Honolulu, HI 96817, USA; E-Mail: alan_white@tnc.org

**Keywords:** marine protected areas, marine reserves, fishing-induced traits, microevolution, Philippines, fisheries, overfishing, conservation

## Abstract

As a consequence of intense fishing pressure, fished populations experience reduced population sizes and shifts in body size toward the predominance of smaller and early maturing individuals. Small, early-maturing fish exhibit significantly reduced reproductive output and, ultimately, reduced fitness. As part of resource management and biodiversity conservation programs worldwide, no-take marine protected areas (MPAs) are expected to ameliorate the adverse effects of fishing pressure. In an attempt to advance our understanding of how coral reef MPAs meet their long-term goals, this study used visual census data from 23 MPAs and fished reefs in the Philippines to address three questions: (1) Do MPAs promote shifts in fish body size frequency distribution towards larger body sizes when compared to fished reefs? (2) Do MPA size and (3) age contribute to the efficacy of MPAs in promoting such shifts? This study revealed that across all MPAs surveyed, the distribution of fishes between MPAs and fished reefs were similar; however, large-bodied fish were more abundant within MPAs, along with small, young-of-the-year individuals. Additionally, there was a significant shift in body size frequency distribution towards larger body sizes in 12 of 23 individual reef sites surveyed. Of 22 fish families, eleven demonstrated significantly different body size frequency distributions between MPAs and fished reefs, indicating that shifts in the size spectrum of fishes in response to protection are family-specific. Family-level shifts demonstrated a significant, positive correlation with MPA age, indicating that MPAs become more effective at increasing the density of large-bodied fish within their boundaries over time.

## 1. Introduction

Overfishing has long been known to adversely affect fish population dynamics, the most notable consequence of which is the precipitous decline in the population size of exploited species [1,2,3,4,5]. The threat of losing livelihood in coastal communities worldwide as a consequence of reduced resource (*i.e.*, fish and invertebrate) biomass, and perhaps local extinction, has motivated governments to establish marine reserves or marine protected areas (MPAs) to help manage marine coastal ecosystems and fishery resources [6,7]. Marine protected areas are subsets of coastal ecosystems (principally coral reefs) designated as no fishing or regulated fishing zones, established with the intent of ensuring the sustainability of marine fisheries and maintaining high biodiversity in marine ecosystems [8,9,10,11]. 

Assessments of MPA efficacy have historically been conducted via comparisons of fish density between MPAs and fished reefs or within an individual reef before and after MPA establishment [12,13]. Recent research, however, has indicated that fish biomass and diversity [14,15,16], as well as reproductive capacity [8] are significantly increased by MPAs and that such metrics are more indicative of true reserve efficacy [17]. Although these approaches yield useful information on the performance of MPAs, they provide little insight into the long-term and, especially, evolutionary importance of marine conservation programs that may be more indicative of long-term reserve efficacy. 

Intense fishing pressure induces significant evolutionary changes in harvested populations [18,19,20,21]. Whether due to selective fishing or legal size restrictions, fishing efforts have an overwhelming tendency to remove the largest individuals from any given population [22,23,24]. Sustained removal of large individuals consequently drives the size distribution of fished populations toward the predominance of smaller individuals (*i.e.*, directional selection) [25,26,27,28] and triggers the onset of sexual maturation in increasingly smaller and younger fish [29,30,31,32]. In fishes, younger females breed for shorter periods, exhibit lower fecundity and produce smaller eggs compared to older conspecifics [33,34,35]. Furthermore, a smaller body size correlates with reduced egg volume, viability, larval size-at-hatch, vertebral number, feeding rate and growth rate [36,37]. These traits greatly reduce the reproductive output and, therefore, fitness of exploited populations and are commonly considered “maladaptive” [37]. Decreased population size compounded by reduced reproductive capacity can cause rapid declines of fish stocks and even result in the total collapse of fisheries, as demonstrated by the near complete disappearance of Atlantic cod off southern Labrador and eastern Newfoundland in the late 1980s [38,39]. Furthermore, a sustained fishing effort imposed upon fish populations by a rapidly growing human population dependent on fisheries is likely to drive evolutionary changes in contemporary time scales [21]. In a period of accelerating marine resource exploitation, understanding how such evolutionary processes affect exploited populations is essential for evaluating the efficacy of marine conservation and fishery management strategies [40,41]. 

By removing fishing pressure, MPAs are expected to halt directional evolution towards smaller body size and the early onset of maturation and should, therefore, arrest the development of maladaptive traits in recovering populations [42,43,44]. The development of maladaptive traits may even be reversed by protection, as ecological forces induce directional selection pressures favoring larger body size [45]. Laboratory manipulations have demonstrated the capacity of fish populations to reverse the development of maladaptive traits in as few as six generations, with the full recovery of historical body size distributions occurring around the 12th generation [46]. However, such shifts likely occur much slower in wild populations, due to the mitigating effects of predation and habitat degradation [14,27]. The ability of MPAs to induce such changes in natural settings, however, has yet to be accurately determined. 

In an attempt to initiate investigations concerning how MPAs achieve their long-term (evolutionary) goals in coral reef ecosystems, this study was designed to address three questions: (1) Do MPAs promote shifts in fish body size frequency distributions towards larger body sizes when compared to fished reefs; and do MPA (2) size and (3) age contribute to the efficacy of MPAs in promoting such shifts? To address these questions, fish body size distributions of coral-reef associated fishes were assessed in 23 no-take coral reef MPAs and fished reefs in the Philippines. Hereafter, all references to “MPAs” or “marine reserves” will refer specifically to no-take protected zones. Body size frequency distributions were analyzed at hierarchical levels to address eight hypotheses:

1. The body size frequency distributions of all fishes censused will be significantly different between MPAs and fished reefs.
2. The body size frequency distributions of fishes between individual MPAs and fished reefs will be significantly different.
3. The occurrence of significant differences in fish body size frequency distributions between MPAs and fished reefs will be associated with MPA size.
4. The occurrence of significant differences in fish body size frequency distributions between MPAs and fished reefs will be associated with MPA age.
5. Fish censused within individual fish families will demonstrate significantly different densities between MPAs and fished reefs.
6. The body size frequency distributions of individual reef fish families will be significantly different between individual MPAs and fished reefs.
7. The size of MPAs will be correlated with the number of fish families demonstrating significant differences in body size frequency distribution between MPAs and fished reefs.
8. The age of MPAs will be correlated with the number of fish families demonstrating significant differences in body size frequency distribution between MPAs and fished reefs.

## 2. Experimental Section

**Figure 1:**
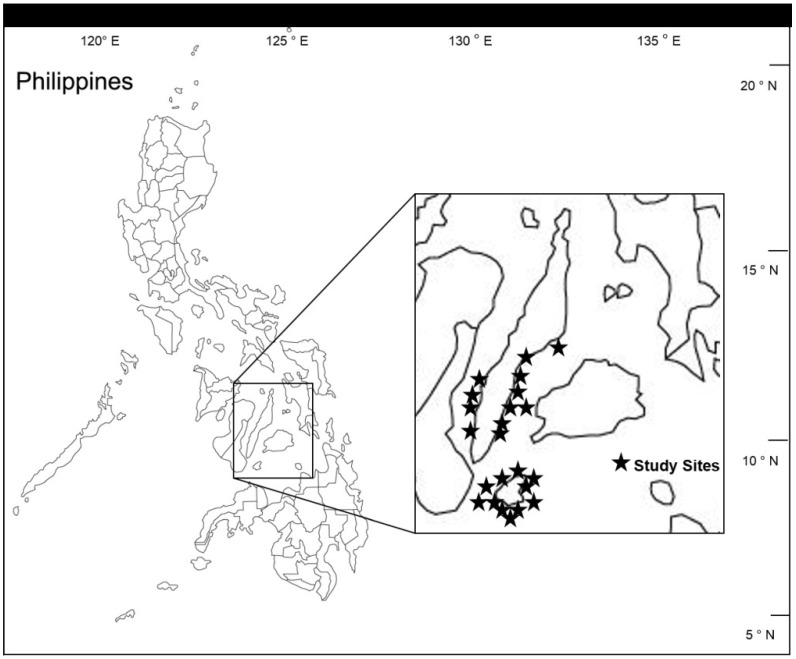
The location of the 23 marine protected areas (MPAs)/fished reef pair sites in Cebu and Siquijor Provinces, Philippines.

To compare the size frequency distribution of fishes between MPAs and fished reefs, fish-visual censuses were conducted along 50 × 10 m belt transects following established protocols [47] in 23 coral reefs in the central Philippines as part of The Coastal Conservation and Education Foundation, Inc. (CCEF) yearly monitoring from 2005 to 2009 (Figure 1). At each reef site, 3–5 transects were set along the reef tract both within the MPA and in fished reefs adjacent to the MPA. Fishes observed in each transect were counted and classified to the family level (22 families total (including sub-families) (Table 1)). Furthermore, each fish was visually assessed for size and placed into one of four size classes (1–10 cm; 11–20 cm; 21–30 cm; >30 cm). 

During the 2005–2009 sampling period, the number of repeated censuses conducted at each site varied from 2–5 sampling years per reef. To account for the disproportionate number of censuses at each individual reef site, the maximum reported density of fish per size category per family among all years surveyed was used to represent fish density in statistical analyses concerning fish body size distributions.

### 2.1. Overall Effects of MPA Protection

To test the hypothesis that the size frequency distribution of all fishes is different between MPAs and fished reefs, the difference in the size frequency distribution of all fishes between all 23 MPAs and fished reefs using the median density of fish (*i.e.*, the median of the maximum fish density of all fishes in each reef among all 23 reefs in each size category) was analyzed using a Kolmogorov-Smirnov (K-S) test of frequencies [48]. Additionally, the difference in median fish density between MPAs and fished reefs in each size class was compared using individual Mann-Whitney U tests [48]. 

**Table 1:** The list of all fish families and sub-families included in the fish visual censuses conducted at all 23 MPAs/fished reef pairs in the Philippines.

Taxon	Common Name
Acanthuridae	Surgeonfish, Tangs
Balistidae	Triggerfish
Bumphead	Bumphead Parrotfish
Caesionidae	Fusiliers
Carangidae	Jacks
Chaetodontidae	Butterfly Fish
Haemulidae	Grunts
Kyphosidae	Chubs, Rudderfish
Labridae	Wrasses
Lethrinidae	Emperor Breams
Lutjanidae	Snappers
Mullidae	Goatfishes
Napoleon	Humphead Wrasses
Nemipteridae	Threadfin Breams
Pomacanthidae	Angelfish
Pomacentridae	Damselfish, Clownfish
Scaridae	Parrotfish
Serranidae	Groupers, Basses, Basslets
Epinephelinae	Serranid Sub-family
Anthiinae	Serranid Sub-family
Siganidae	Rabbitfish
Zanclidae	Moorish Idol

### 2.2. Effects of Individual MPAs

In order to test the hypothesis that the size frequency distribution between fishes within individual MPAs and fished reefs will differ, the total maximum reported densities of all fishes within each size category were compared between each individual MPA and its corresponding fished reef using K-S tests. To test the hypothesis that MPA size and age are associated with the occurrence of significant shifts in fish body size within MPAs, row by column (RxC) contingency table analyses were conducted on the number of significant size shifts and either MPA size or age. 

### 2.3. Effects of Protection on Fish Families across MPAs

To determine whether the densities of reef fish families significantly differed between MPAs and fished reefs, the maximum recorded density of each fish family at each size class between all MPA/fished reef pairs was compared using Scheirer-Ray-Hare (SRH) extensions of a two-factorial Kruskal-Wallis test, with site and size class as the main effects [48]. For families that demonstrated significant effects of protection from SRH analysis, individual Mann-Whitney U tests were performed within each size class to determine which specific size classes differed in densities between MPAs and fished reefs.

### 2.4. Effects on Individual Fish Families

Finally, to test the hypothesis that individual fish families demonstrate significant differences in body size frequency distribution within individual MPA/fished reef sites, the body size frequency distributions of individual fish families between MPAs and fished reefs were compared using K-S tests, totaling 506 tests (23 MPAs/fished reef pairs ×22 fish families). In addition, to determine whether MPA size or age is correlated with the number of significant disparities in body size distribution between MPAs and fished reefs, the number of significant family-level results (*i.e.*, the number of fish families within an individual MPA/fished reef pair that demonstrated a significant shift in body size frequency distribution towards larger body sizes) found at each reef site was plotted against the size and age of each MPA using Spearman rank correlations [48]. 

## 3. Results and Discussion

### 3.1. Overall Effects of MPA Protection

**Figure 2:**
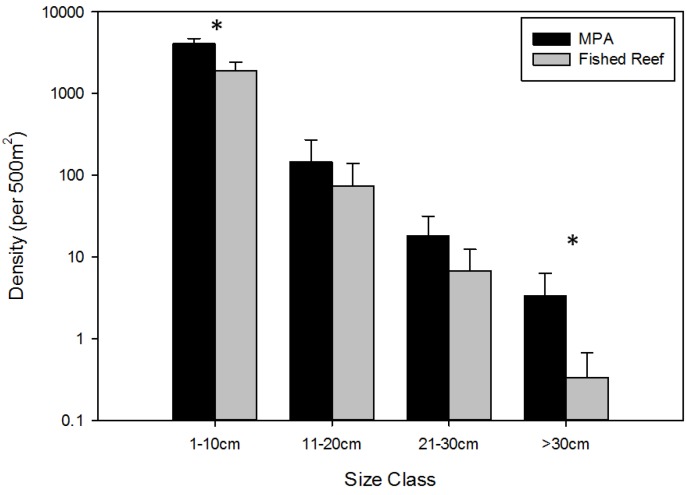
Median ± median absolute deviation (MAD) of the maximum reported densities of all fishes from all censused MPAs. Black bars indicate fish density within MPA borders, and grey bars represent fish density of fished reefs. Asterisks (*) above groups of black and grey bars indicate a significant difference (*p* < 0.001) from individual Mann-Whitney U tests.

In both MPAs and fished reefs, the maximum density of fishes decreased as fish body size increased (Figure 2). Although the difference in the size frequency distribution of all fishes between MPAs and fished reefs was not significant (K-S test: D (test statistic) = 0.00187 Dα = 0.03715; *p* > 0.05), fish density was higher inside MPAs than in fished reefs in all size classes (median density per 500 m^2^ ± median absolute deviation (MAD), (MPAs; fished reefs): 1–10 cm (4070.83 ± 639.10; 1878.00 ± 517.67) ,11–20 cm (146.00 ± 125.67; 74.00 ± 66.00), 21–30 cm (18.00 ± 13.33; 6.67 ± 5.67) and >30 cm (3.33 ± 3.00; 0.33 ± 0.33). Furthermore, in the smallest and largest size classes, Mann-Whitney U tests indicated that fish densities were significantly higher inside MPAs than in fished reefs (1–10 cm: U = 502; *p* < 0.001; 11–20 cm: U = 343; *p* = 0.0865; 21–30 cm: U = 350; *p* = 006174; >30cm: U= 405; *p* < 0.001; N1 = 23, N2 = 23 for all size classes). 

### 3.2. Effects of Individual MPAs

**Figure 3:**
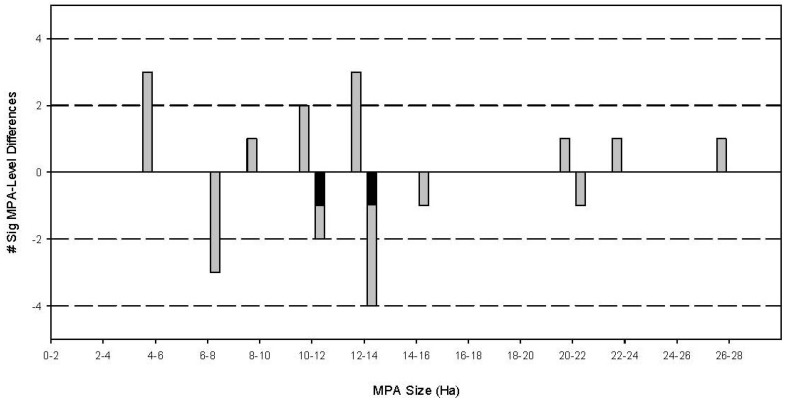
The relationship between the occurrence of a significant body size frequency distribution and MPA size. Grey bars above y = 0 represent the number of MPAs of a given size that demonstrated a significant shift in body size distribution compared to fished reefs. Grey bars below y = 0 represent the number of MPAs of a given size that did not show a significant difference in body size distributions. Black bars below the x-axis represent MPAs that demonstrated a significant shift in body size distributions towards larger body sizes of fishes in fished reefs compared to fishes within MPAs. The results of a contingency table analysis revealed no significant association between MPA size and body size frequency shifts (χ^2^ = 10.1; d.f. = 8; *p* = 0.257).

**Figure 4:**
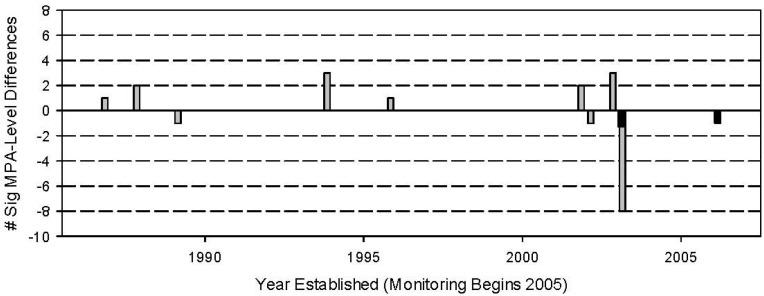
The relationship between the occurrence of significant differences in the body size frequency distribution and MPA age. Grey bars above y = 0 represent the number of MPAs of a given age that demonstrated a significant shift in body size distribution compared to fished reefs. Grey bars below y = 0 represent the number of MPAs of a given age that did not show a significant difference in body size distributions. Black bars below the x-axis represent MPAs that demonstrated a significant shift in body size distributions towards larger body sizes of fishes in fished reefs compared to fishes within MPAs. Results of a contingency table analysis revealed no significant association between MPA size and body size frequency shifts (χ^2^ = 11.6; d.f. = 8; *p* = 0.115).

**Figure 5:**
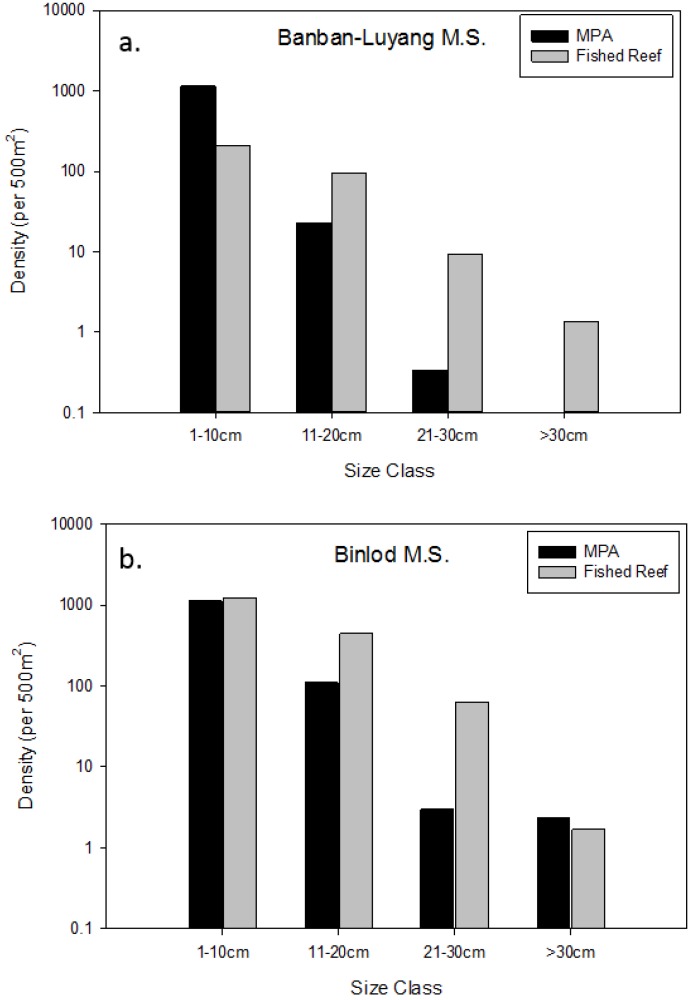
Fish body size frequency distributions from both the (**a**) Banban-Luyang Marine Sanctuary (M.S.) and the (**b**) Binlod Marine Sanctuary, both of which demonstrated a significant shift towards larger fish body sizes in fished reefs compared to fishes within MPAs. Black bars represent the fish density of fishes inside MPAs, while grey bars represent fish density in fished reefs.

In 12 of 23 MPA-fished reef pairs, there were significant shifts in the body size distribution, wherein large-bodied fishes were more abundant in MPAs compared to corresponding fished reefs (Table 2). The occurrence of significant size shifts spread throughout the range of MPA size and age suggests that these traits did not influence the development of differential fish body size frequency distributions (Figure 3 and Figure 4). This was confirmed by contingency table analyses (MPA size: χ^2^ = 10.1; degrees of freedom (d.f.) = 8; *p* = 0.257; MPA age: χ^2^ = 11.6; d.f. = 8; *p* = 0.115). Of the four MPAs established in 1987–1989, the oldest in this study, three (Tulapos Marine Sanctuary (M.S.) (established 1987), Taculing-Cangmalag M.S. (established 1988) and Olang M.S. (established 1988)) had significantly higher densities of fish in larger body size categories within their borders than in fished reefs. Surprisingly, three of the most recently established MPAs in this study (Sandugan M.S., Lower-Cabangcalan M.S. and Daang-Lungsod M.S. (all established 2003)) demonstrated significant shifts in fish body size distribution towards larger body sizes compared to fished reefs, as well. Similarly, only two of the three largest MPAs contained significantly larger fish than in fished reefs (Tulapos M.S. (27.22 ha), Daang-Lungsod M.S. (22.71 ha)), while the smallest MPA in the study (Casay Shoal M.S. (5.00 ha)) also demonstrated significant increases in fish body size distribution within its borders. Interestingly, the occurrence of fish size shift was not always as consistent as would be expected. In two MPAs, Banban-Luyang Marine Sanctuary (established 2006; size: 10 ha) and the Binlod Marine Sanctuary (established 2003; size: 12 ha), the significant shifts in body size distribution were driven by the presence of more fishes in the larger size categories in fished reefs compared to MPAs (Figure 5).

**Table 2:** Results of Kolmogorov-Smirnov tests on maximum reported densities of all fishes between MPA borders and fished reefs in each of the 23 MPAs surveyed. Tests were conducted at an alpha level (α) of 0.05. Asterisks (*) indicate that a reserve demonstrated a significant shift towards larger body sizes in fished reefs compared to fishes within MPAs.

Marine Protected Area (Associated Province)	Year Established	Size (ha)	Dα	D	Significance (D > Dα)
Tulapos (Siquijor)	1987	27.22	0.0416	0.2489	YES
Taculing-Cangmalalag (Siquijor)	1988	13.38	0.0840	0.1964	YES
Olang (Siquijor)	1988	21.36	0.0406	0.0680	YES
Tobod (Siquijor)	1989	7.5	0.0329	0.0096	NO
Caticugan (Siquijor)	1994	13.51	0.0511	0.0907	YES
Madridejos (Cebu)	1994	10.78	0.0474	0.0947	YES
Sta Filomena (Cebu)	1994	5.62	0.0389	0.0391	YES
Nonoc (Siquijor)	1996	5.75	0.0417	0.1327	YES
Poblacion Alcoy (Cebu)	2002	6.38	0.0492	0.0102	NO
Legaspi (Cebu)	2002	10.35	0.0578	0.1916	YES
Casay Shoal (Cebu)	2002	5	0.0345	0.0636	YES
Sandugan (Siquijor)	2003	13.38	0.0413	0.0879	YES
Lower-Cabangcalan (Siquijor)	2003	8.23	0.0493	0.1021	YES
Talayong (Siquijor)	2003	6.68	0.0425	0.0133	NO
Bogo (Siquijor)	2003	10	0.0503	0.0238	NO
Candaping B (Siquijor)	2003	20.42	0.0529	0.0210	NO
Minalulan (Siquijor)	2003	14.63	0.1630	0.0511	NO
Daang-Lungsod (Cebu)	2003	22.71	0.0272	0.1018	YES
Binlod (Cebu)	2003	12	0.0507	0.1996	YES *
Bogo (Cebu)	2003	12	0.0468	0.0216	NO
Bulasa (Cebu)	2003	12	0.0484	0.0427	NO
Guiwanon (Cebu)	2003	12	0.0740	0.0035	NO
Banban-Luyang (Siquijor)	2006	10	0.0494	0.3175	YES *

### 3.3. Effects of Protection on Fish Families across MPAs

Fish density differed between MPA and fished reefs in five of 22 fish families (Scheirer-Ray-Hare extensions of the Kruskal-Wallis test: Lutjanidae (H: 40.363; d.f.: 7; *p* < 0.001), Haemulidae (H: 33.385; d.f.: 7; *p* < 0.001), Scaridae (H: 93.903; d.f.: 8; *p* < 0.001), Acanthuridae (H: 75.197; d.f.: 7; *p* < 0.001) Epinephelinae (H: 70.804; d.f.; 7; *p* < 0.001)). Individual Mann–Whitney U tests revealed that although significant increases in density first appeared in a variety of size classes (Table 3), two trends were evident: (1) four of the five families demonstrated significantly higher densities of fish in the largest size class (>30 cm); and (2) with the exception of Scaridae and Lutjanidae, after the first appearance of a significant increase in density within an individual size class, all larger size classes also demonstrated a significant increase in density.

**Table 3:** Results of post-hoc Mann-Whitney U tests on the five families that demonstrated a significant difference in densities in Kruskal-Wallis tests. Bold *p*-values indicate significantly higher densities of fishes within MPAs compared to fished reefs in a given size class.

Family	1–10 cm			11–20 cm			21–30 cm			>30 cm		
	N	U	*p*	N	U	*p*	N	U	*p*	N	U	*p*
Lutjanidae	46	151	**0.006**	46	156.5	**0.014**	46	178	**0.038**	46	213.5	0.09
Haemulidae	46	212	0.211	46	190	0.076	46	181	**0.014**	46	218.5	**0.039**
Scaridae	46	240.5	0.598	46	160	**0.022**	46	178	0.057	46	147.5	**0.008**
Acanthuridae	46	236.5	0.538	46	162.5	**0.025**	46	172	**0.031**	46	161	**0.001**
Epinephelinae	46	217	0.294	46	181.5	0.62	46	217.5	0.193	46	187	**0.014**

### 3.4. Effects on Individual Fish Families

**Figure 6:**
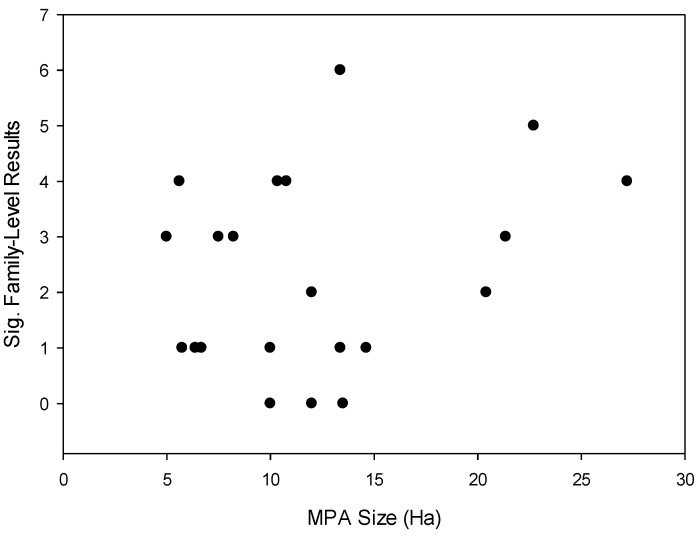
Scatter plot of the occurrence of statistically significant disparities in fish body size distribution between MPAs and fished reefs across MPAs of varying size (ha), based on the results of family-level K-S tests. A Spearman rank correlation revealed no association between the two variables (rs = 0.110; *p* = 0.614).

**Figure 7:**
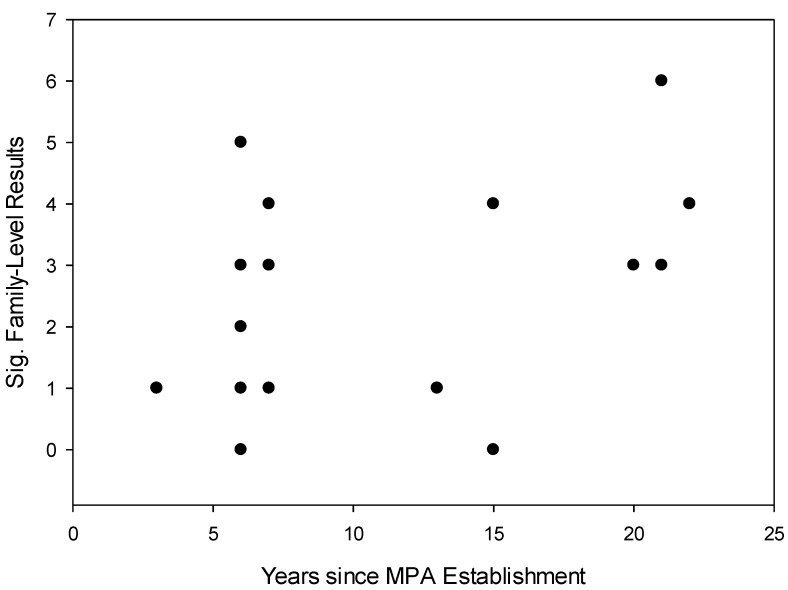
Correlation between the occurrence of statistically significant disparities in fish body size distribution between MPAs and fished reefs across MPAs of varying age, based on the results of family-level K-S tests. A Spearman rank correlation indicated a significant, positive association between the two variables (rs = 0.500; *p* = 0.0153).

Eleven of the 22 families exhibited a shift in fish body size distribution, with more large-bodied Pomacentridae, Scaridae, Caesionidae, Acanthuridae, Anthiinae, Labridae, Balistidae, Chaetodontidae, Lutjanidae, Mullidae and Siganidae within MPAs compared to fished reefs. However, some families of fishes had more large-bodied fish outside MPAs compared to corresponding sites within MPAs. These include Caesionidae, Acanthuridae, Scaridae, Chaetodontidae, Mullidae, Nemipteridae and Pomacentridae. The occurrence of a body size shift was not affected by MPA size (r_s_: 0.110; *p* = 0.614) (Figure 6); however, there was a positive, highly significant correlation between the age of MPAs and the number of family-level shifts within individual MPAs (r_s_: 0.500; *p* = 0.0153) (Figure 7). These results indicate that although the size of individual MPAs does not appear to play a significant role in MPA efficacy, the efficacy of MPAs increase over time, as older MPAs have facilitated shifts in body size towards larger fish in more families than younger MPAs.

Philippine coral reefs are nestled in the northern region of the Coral Triangle, an area of roughly 5.7 million square kilometers that contains some of the highest marine biodiversity on the planet, containing over 3000 fish and 600 coral species [49]. The marine resources within the Coral Triangle sustain 120 million people, over two million of which are fishermen [50]. Accelerated human population growth in the triangle, however, has put extreme pressure on fish populations. Increasing and intensive fishing pressure has not only resulted in the reduction of biological diversity within exploited marine ecosystems, but also augmented hardships among local resource users. As MPAs increase in popularity as a primary tool for marine conservation in the region, it is essential that the efficacy of MPAs in ameliorating the adverse effects of fishing pressure is accurately assessed. 

Although there was no difference in the body size frequency distributions of fishes between MPAs and fished reefs across all reefs surveyed, the predominance of large-bodied (>30 cm) fishes inside MPAs suggests the development of a directional shift in body size towards larger body sizes may be developing with protected areas. Additionally, the significantly higher density of small-bodied fish may be indicative of higher reproductive output predicted by the predominance of large-bodied fish with MPA borders. Increased fecundity over time should cascade into larger body size classes, resulting in directional shifts in the overall body size frequency distribution of fishes in MPAs compared to fished reefs. It is also possible, however, that it is simply not feasible to detect shifts in size distribution at such a large resolution, as large differences in the average body size exist both between and within reef fish families. Investigations into the disparity of size distribution across MPA networks at the species level would be ideal, but sufficient data for such an analysis was not available for this study.

Within families that demonstrated a significant effect of reserve protection, increases in density were skewed towards large size classes, most notably the largest size class (>30 cm), in which all families, except Lutjanidae, had significantly higher densities within MPAs. Furthermore, with the exceptions of Scaridae and Lutjanidae, increases in density within families continued at increasing size classes. Such a result is interesting for two reasons. Firstly, although intra-familial variation in body size exists, it is significantly lower than variation between families. Secondly, the continuing disparity of densities within increasing size classes indicates that not only are there more fishes within MPAs compared to fished reefs, but that fish inside MPAs reach larger sizes compared to their conspecifics in fished reefs. Family-specific response to protection, most specifically in Scaridae, Acanthuridae [51] and Caesionidae [52], may be attributed to family-specific susceptibility to fishing pressure [52] and recovery rates [53] following exposure to intense fishing pressure. It is hypothesized that the disparity in size frequency distribution between MPAs and fished reefs will increase over time, as populations in fished reefs are continually subjected to fishing pressure, resulting in further directional selection favoring smaller fish body size. Other factors that may contribute to the variation in the occurrence of fish size frequency shifts between fished reefs and MPAs include: reserve habitat (particularly the percentage of hard coral cover and habitat complexity); the proximity to coastline and pollutant runoff; as well as the levels of reserve enforcement [54] and the levels of reserve compliance by local resource users [55]. Additionally, initial stocks at the time of MPA establishment, as well as varying fishing pressures at different sites may play vital roles, especially since recent studies have found increasing use of highly efficient fishing gear (trammel nets, *etc.*) around MPAs in the Philippines, resulting in severe depletion of stocks around MPAs [56,57]. 

It is conceivable that the absence of size frequency differences between MPAs and fished reefs found in this study is the result of equal growth in body size in both populations since the establishment of MPAs in the Philippines. This would be representative of the dual processes of “spillover” and “recruitment subsidy”, in which adults and larvae from MPAs, respectively, augment fishery stocks outside reserve borders [58]. These processes have been observed in numerous MPAs in the Philippines [59,60,61,62] and in other locations worldwide [15]. Unfortunately, no comparable surveys were conducted in any of the MPAs analyzed in this study before their establishment, and therefore, no baseline for which to compare current census data exists. 

The unexpected results showing fish body size distributions skewed towards larger size classes in fished reefs compared to MPAs are particularly intriguing. In newly designated MPAs, it is possible that there has not been enough time for fish populations to recover from overfishing. It is also conceivable that these particular sites were established around suboptimal reefs. With multiple stakeholders influencing the decision of where an MPA is to be placed (conservationists, fisherman, local governmental units, *etc.*), it is often the case that MPAs are placed in sites that are not suitable for the promotion of significant population recovery [62,63].

With the increasing use of MPAs as both conservation and fishery management strategies, it is vital that MPAs are effectively assessed in terms of their ability to reverse the detrimental consequences of severe fishing pressure. To maintain and subsequently increase exploited fish populations, MPAs must successfully reverse the intense directional selection pressure caused by the anthropogenic fishing effort. MPA-induced evolutionary changes that restore historical fish body size distributions, along with local and regional conservation efforts to combat external influences (including water pollution, natural resource exploitation, development and extreme overfishing outside of reserve borders), have the potential to restore overharvested populations to sustainable levels [64,65]. The recovery of fish populations is not only vital to the ecosystems in which these fishes reside, but also to the sustainability of the livelihood of millions of people who rely on marine resources for survival. A combination of effective management strategies will result in the recovery of severely exploited fishery stocks worldwide, thus preserving invaluable sources of biological diversity and resources. Without sufficient protection, however, overexploited populations are likely to be pushed to the point of complete collapse in coming decades. If marine protected areas are not capable of combating the negative evolutionary repercussions of highly exploitative fishing pressure, current management strategies may need to be reexamined and redesigned to more effectively protect both a natural resource and a source of incredible biodiversity for future generations [66].

## 4. Conclusions

Comparisons of the body size frequency distribution of fishes between MPAs and fished reefs in 23 coral reefs in central Philippines revealed that: (1) accounting for all fishes observed, there were significantly more fishes within the largest and smallest size classes within MPAs compared to fished reefs; (2) only specific reef fish families exhibited shifts in the size frequency distribution, with a higher frequency of occurrence of large size classes relative to smaller-sized confamilials within MPAs compared to fished reefs; (3) the occurrence of body size shifts are widespread among MPAs of varying size, and (4) there was a significant correlation between the age of MPAs and the number of fish families positively responding to protection within their borders. These results are consistent with previous studies that have demonstrated increased biomass within reserve borders compared to fished reefs [8,15,51].

In the context of microevolutionary processes, current understanding of how MPAs contribute to the conservation of marine biodiversity and the sustainability of marine fisheries resources is highly limited. Field-based analyses of the evolutionary responses of fishes to protection are a rarity, due to the relatively recent development of the understanding of such processes and the difficult nature of evolutionary investigations on wild populations. Although size spectrum analyses, such as the one conducted here, will be vital in identifying regions in which MPAs have resulted in phenotypic shifts in protected populations, further research is required to more fully understand the evolutionary implications of MPAs on exploited populations and the underlying processes driving shifts in body size frequency distributions.
